# Clinical comparison of two automated audiometry procedures

**DOI:** 10.3389/fnins.2022.1011016

**Published:** 2022-10-11

**Authors:** Hui Liu, Bingqing Du, Bo Liu, Xinxing Fu, Yao Wang

**Affiliations:** ^1^Department of Otolaryngology, Head and Neck Surgery, Beijing Tongren Hospital, Capital Medical University, Beijing, China; ^2^Beijing Institute of Otolaryngology, Beijing, China; ^3^Medical School, The University of Western Australia, Crawley, WA, Australia; ^4^Ear Science Institute Australia, Subiaco, WA, Australia; ^5^School of Life Sciences, Tiangong University, Tianjin, China; ^6^School of Precision Instruments and Optoelectronics Engineering, Tianjin University, Tianjin, China

**Keywords:** automated audiometry, audiometry, KUDUwave, ascending method, shortened ascending method

## Abstract

**Objective:**

Automated pure-tone audiometry has been shown to provide similar hearing threshold estimates to conventional audiometry, but lower correlations were reported at high and low frequencies in audiometric tests than those of manual tests, while the correlations were better in the middle frequencies. In this paper, we used the same equipment and different test procedures for automated testing, and compared the results with manual test results.

**Design:**

One hundred subjects aged 18–36 years were randomly divided into two groups to perform air-conduction pure-tone audiometry (0.25, 0.5, 1, 2, 4, 8 kHz) using the ascending and shortened ascending protocols built-in to the automated audiometer, respectively. Recorded testing time, the total number of responses and the subject’s preference tests were compared with those of manual tests.

**Results:**

Significant difference was found at 250 Hz regarding the distribution of the absolute difference between the two automated and the manual thresholds. The testing time spend in the ascending method (9.8 ± 1.4 min, mean ± SD) was significantly longer than in the shorted ascending method (5.8 ± 0.9 min). The total numbers of responses of the ascending method (90.5 ± 10.8 times) and shorted ascending method (62.0 ± 11.4 times) were significantly different. Finally, no significant difference was found in preferences between automated and manual procedures.

**Conclusion:**

The shorted ascending method can save lots of testing time. The difference between the two automated thresholds at 250 Hz is caused by the different test procedures, and the difference at 8,000 Hz between the automated test and the manual test can be due to the transducer types and allowable differences in calibration.

## Introduction

More than 1.5 billion people worldwide are living with some degree of hearing loss, equivalent to 20% of the total population. At least 430 million of them have moderate or higher levels of hearing loss, also known as disabling hearing loss (World Health Organization [WHO], 2021). In China, according to the results of the Second National Sample Survey on Disability, 27.8 million people had hearing disabilities, of which 20.04 million people were suffering from hearing disability alone and 7.76 million were suffering from multi-disabilities (Xi-bin et al., 2008). Given the large number of people with hearing loss, China is experiencing an extreme lack of experts who can provide high-quality hearing services (Chadha et al., 2021). Currently, only 10,000 audiologists provide hearing services for 1.37 billion people in China (1:137000) (Chung et al., 2014).

Pure-tone audiometry is the gold standard for a clinical hearing assessment. For many years, pure-tone audiometry has always relied on traditional manual audiometry. However, pure-tone audiometry is a test based on sequence and step inspection, which is particularly suitable for the automation (Margolis and Morgan, 2008). Automation is a powerful enabler for alternative diagnostic pathways, which can reduce testing costs without trained audiologists (Eksteen et al., 2019) and testing outside a sound booth (Brennan-Jones et al., 2016; Magro et al., 2020), and potentially benefit people with hearing loss in remote and economically underdeveloped areas (Visagie et al., 2015; Sandström et al., 2020), to address the global need for an accessible hearing loss diagnosis (Swanepoel et al., 2019; Sidiras et al., 2021; Wasmann et al., 2022).

There is a high correlation between the manual and automated pure-tone audiometry, with an overall average difference of 0.4 ± 6.1 dB regarding the air conduction threshold (Mahomed et al., 2013). However, lower correlations in automated thresholds at high (6,000 or 8,000 Hz) and low (250 or 500 Hz) frequencies were reported in previous studies. Compared with the standard methods, the threshold difference varied from 8.7 to 17 dB at the low or high-frequency points, significantly higher than those at overall frequencies (Abu-Ghanem et al., 2016; Brennan-Jones et al., 2016; Corry et al., 2017; Sandström et al., 2020). In the studies of automated audiometry, the explanation of this phenomenon varied due to different testing environments and different types of equipment. The differences in low frequencies were interpreted as the effect of ambient noise levels and suboptimal fitting of the earphones (Abu-Ghanem et al., 2016; Corry et al., 2017), whereas the differences in high frequencies were susceptible to variations in the coupling of headphones or earphones and individual physiological differences (Brennan-Jones et al., 2016; Corry et al., 2017; Sandström et al., 2020). However, there are no reports on whether such variability is due to the difference in automated audiometry procedures.

There are three methods for automated measurement of pure-tone thresholds: the automated method of adjustment, the automated method of limits, and the automated adaptive method (Jerger, 2018). According to a scoping review, in the last decade, about 74% of published studies on automated tests utilized the modified Hughson-Westlake procedure, which is based on the classical method of limits (Wasmann et al., 2022). Two kinds of threshold-seeking procedures were recommended in the modified Hughson–Westlake protocol by ISO 8253-1:2010, i.e., ascending and shortened ascending procedures. The ascending procedure stipulated that when three reactions occurred at the same test sound level during a maximum of five ascents, then this sound level was determined as the hearing threshold level. For the shortened ascending method, the hearing threshold level was identified as at least two reactions that occurred at the same level out of three ascents. In the current literature, some automated tests use the shortened method, and some studies do not specify the test method. In ISO 8253-1-2010, it is stated that the shortened and ascending method can obtain almost identical results. The guidelines do not state whether the same consistent results can be obtained when using both methods for automated testing. In manual testing, the shortened method can be used for special subjects, such as those who cannot concentrate for long periods of time, where the test time is more important than the reliability of the threshold.

In clinical testing, the subject’s response profile is complex and variable. During manual testing, an experienced audiologist will make observations of the subject’s behavior. Whether the ascending or the shortened ascending method is used, the audiologist will guarantee the reliability of the test results. However, in the programmed automated test, although the correlation between the results of the automated and the manual test, in terms of the overall (average of the hearing thresholds for all frequencies), was good; the correlation was poor in the lower and higher frequencies. There are no clinical data on whether the use of the shortened ascending method in automatic testing will sacrifice reliability at certain frequencies. In this study, two automated audiometry protocols, ascending and shortened ascending methods, were used to compare the results with manual audiometry, respectively, to observe the correlation between the two methods at all testing frequencies and to investigate the effect of different automated methods on the test results.

## Materials and methods

### Subjects

One hundred normal hearing participants (56 females) ranged 18–36 years (median age was 27 years) from the Otolaryngology Clinic of Beijing Tongren Hospital were recruited in this study. The inclusion criteria were: (World Health Organization [WHO], 2021) aged 18 years or above, (Xi-bin et al., 2008) no known cognitive disorder, (Chadha et al., 2021). Mandarin as a first language, (Chung et al., 2014) four-frequency average (500, 1,000, 2,000, and 4,000 Hz) air-conduction thresholds of both ears ≤15 dB HL, (Margolis and Morgan, 2008) normal otoscope examination. This study was approved by the Ethics Committee of Beijing Tongren Hospital, Capital Medical University. The participants all provided written informed consent before the test.

### Equipment

The clinical diagnostic audiometer (Otometrics Conera) was used for the manual pure-tone hearing threshold test with TDH-39 supra-aural earphones. The calibration was conducted according to ISO 389-1: 2017. Automated audiometry was conducted using the KUDUwave (GeoAxon, Pretoria, South Africa) audiometer, which used insert earphones for air conduction thresholds testing. The circumaural headphones of KUDUwave are placed above the insert earphones to increase the attenuation of ambient sound, meanwhile, the audiometer monitors background noise levels *via* an external microphone (outside of the circumaural headphone cup) and an internal microphone (inside of the circumaural headphone cup) to ensure testing compliance (Swanepoel de et al., 2010). As noted by Storey et al. (2014), with this combination of attenuation and monitoring, patients can be reliably tested to −10 dB HL at 55 dB ambient noise and to 0 dB HL at 70 dB ambient noise. The KUDUwave was connected to the computer through the USB port, and the test process was controlled by the software installed in the notebook computer. The KUDUwave was measured and calibrated before use in accordance with ISO 389-2: 1994. All tests were carried out in an American National Standards Institute (ANSI) certified double-walled sound-treated booth.

### Test methods

The otoscope examination, tympanic admittance measurement and manual air conduction hearing threshold test were performed for all participants. The participants who met the inclusion criteria were numbered in the order of 1–100, the participants with odd numbers were tested by the ascending program (Group A) for automated testing, and even-numbered participants were tested by the shortened ascending program (Group S) for automated testing.

The manual audiometry was conducted by an audiologist with at least 30 years of testing experience. The test requirements were fully explained to the participant before the test. The participants were asked to quickly press and release the response button whenever the tone is heard in either ear, no matter how faint it may be. After the participants fully understood the test requirements, they wore air conduction headphones, and the hearing thresholds were determined according to the standard clinical procedure (modified Hughson–Westlake, ISO 8253-1). The test frequency was at octave frequencies from 250 to 8,000 Hz.

The automated test process was completed by an undergraduate student in audiology. The participants were informed of the test process and requirements, which were the same as the manual test. Insert earphones were deeply inserted and the end of the insert foam tips were flush with the opening of the external auditory meatus. The circumaural earcups of KUDUwave were placed over insert earphones to increase the attenuation of environmental sound, and ensured comfort and stability. The conditioning page interface was presented to play stimulus to the participant and observe the response time. After the subject fully understood the test requirements, the automated test was started to determine the hearing thresholds. The test frequency was at octave frequencies from 250 to 8,000 Hz.

The ascending and shortened ascending procedures of the KUDUwave automated test program were adopted. The initial intensity of each frequency was 30 dB HL, and the sound duration lasted for 1,000 ms. A valid response was considered as pressing the response button within 2,500 ms after delivering the pure tone, or it will be marked as a false positive response by KUDUwave. After the test, KUDUwave automatically reports the percentage of false positives, the number of times the subject responded to the pure tone and the response time the subject pressed the response button after the pure tone is delivered. A detailed description of the automated and manual protocols was listed in the (Supplementary Table 1).

The test time required for manual testing and automated testing was manually recorded and compared. The time for explaining test requirements, wearing headphones, and familiarizing with the sound test process were not included in the recorded test time. The participants were asked about their preference for manual and automated testing methods after the test finished, preferred the automated test, preferred the manual test, or had no preference. To avoid differences in the background noise of the test environment from affecting the test results, all manual and automated tests were conducted in one sound booth.

### Data processing

Descriptive measures illustrated the difference between the thresholds of manual and automated audiometry, described as mean ± SD. The test time required for manual and automated audiometry and the total number of reactions were described as mean ± SD. The preference for the test methods was described as a percentage. A paired *t*-test was employed across the frequencies of 250–8,000 Hz to test whether there is a significant difference between the thresholds of manual and automated audiometry. Comparisons between Groups A and S were evaluated using independent *t*-tests, including the testing time and the total number of reactions. The chi-squared test is used to determine whether there is a significant difference in the distribution of the difference of threshold between Groups A and S. The chi-squared test is also used to test the difference in the participants’ preference for two automated procedures. An ANOVA test was conducted to test the effect of gender and age on the thresholds of the pure tone audiometry. All statistical analyses were performed by SPSS 25 (SPSS Inc., Chicago, IL, USA). A *p*-value of less than 0.05 was considered statistically significant.

## Results

In order to compare the automated and manual test results, the difference values were calculated between clinical audiometer thresholds and the KUDUwave thresholds for the two automated procedures. As shown in Table 1, the two automated test methods were more accurate at the range of 500 to 4,000 Hz, while the accuracy at 250 and 8,000 Hz were poor. Nevertheless, the automated thresholds at all frequencies had a good correlation with the manual thresholds at all frequencies.

**TABLE 1:** The difference and correlations between manual and automated audiometry thresholds.

Hz	250	500	1,000	2,000	4,000	8,000
**Ascending method**						
M difference in dB (SD)	−3.52 (4.77)	−0.46 (5.08)	0.61 (4.14)	−0.46 (5.18)	2.04 (4.97)	5.61 (6.27)
Abs M difference in dB (SD)	4.03 (4.35)	3.32 (3.86)	2.76 (3.14)	3.83 (3.50)	4.08 (3.47)	6.63 (5.17)
Correlations	0.88	0.89	0.96	0.95	0.96	0.95
**Shortened ascending method**						
M difference in dB (SD)	−4.26 (4.12)	−1.02 (5.09)	−0.17 (4.32)	−0.68 (5.42)	0.80 (5.14)	6.99 (7.49)
Abs M difference in dB (SD)	4.94 (3.26)	3.75 (3.58)	2.78 (3.29)	4.09 (3.60)	3.75 (3.58)	8.01 (6.37)
Correlations	0.91	0.90	0.95	0.95	0.95	0.95

M difference: the average value of the difference between the manual and the automated thresholds (manual minus automated values); Abs M difference: the average of the absolute value of the difference between the manual and the automated threshold; Correlation: the correlation coefficients between manual and automated test results.

**FIGURE 1:**
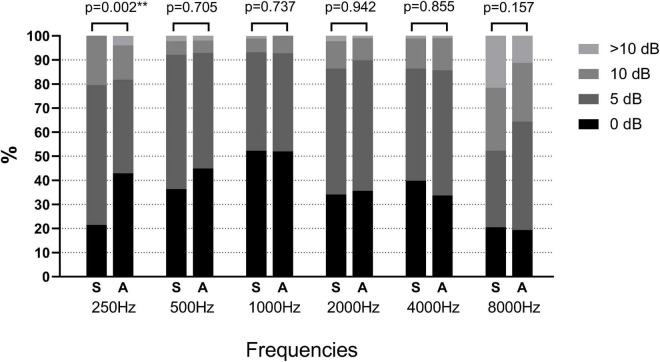
Distribution of the absolute differences between each type of automated test threshold (Groups A and S) and the manual test threshold at each frequency. Asterisk values indicate a statistically significant difference with a *p*-value less than 0.05. S, shortened ascending method; A, ascending method.

The distribution of the absolute difference between the manual and the automated thresholds was shown in Figure 1. Only a significant difference was found at 250 Hz (*p* = 0.002), the number of thresholds difference within 5 dB in Group S was higher than that in Group A, while the number of threshold differences within 0 dB in Group S was less than that in Group A. The correlation of the automated thresholds at 8,000 Hz between the two groups was low, and the percentage compared with the manual test results less or equal to 5 dB was smaller than that at other frequencies. However, no statistical difference was observed between the two groups at 8,000 Hz.

The differences in test time, participants’ preferences, and the total number of reactions between the two automated groups were listed in Table 2. The automated test time in Group S was significantly shorter than in Group A (Group A: 9.8 ± 1.4, Group S: 5.8 ± 0.9, *p* < 0.001). Accordingly, the total number of reactions in Group S was also less than in Group A, because Group S used shortened ascending method. Most of the participants did not favor automated tests, and the main feedback was that the headphones of the automated audiometer were heavier, especially in Group A, because the test time was much longer than in Group S. However, no significant difference existed in preference between automated and manual procedures.

**TABLE 2:** Differences in test time, preference on the testing methods, and the total number of reactions between Groups A and S.

	Testing time (min)	Preference (%)			Total number of reactions
	Automated	Manual	Automated	Whatever	Automated
Group S	5.8 ± 0.9	31.6	21.1	47.4	62.0 ± 11.4
Group A	9.8 ± 1.4	36.7	16.3	46.9	90.5 ± 10.8
*P*-values	*P* < 0.001*		*p* = 0.795		*P* < 0.001*

Asterisk values indicate a statistically significant difference with a *p*-value less than 0.05.

In addition, we analyzed the effect of gender and age on the results of the automated test. According to the ANOVA test, no significant differences between automated and manual audiometry thresholds were found regarding the gender or age of the subjects, the results were listed as (Supplementary Table 1).

## Discussion

In this study, we tested two automated audiometry procedures, ascending and shortened ascending methods, and compared the automated thresholds with manual test results. Similar to previous reports, the automated audiometry correlated well with the manual test. However, we found lower correlation at 250 and 8,000 Hz than at the other frequencies.

Hearing thresholds obtained from two automated procedures showed a greater variation at 8,000 Hz compared to manual tests. There were a larger number of hearing threshold differences of 10 dB or above at 8,000 Hz. Other studies also reported higher mean threshold differences for automated tests at 8,000 Hz than other frequencies (Storey et al., 2014; Brennan-Jones et al., 2016; Barbour et al., 2019; Sandström et al., 2020). The reason for this is likely to be systematic differences in transducer types, and allowable differences in the calibration (Sandström et al., 2020). The use of insert earphones may have introduced additional variation at high frequencies compared to supra-aural headphones (Brennan-Jones et al., 2016). In this study, the insert earphones were used for the automated test and supra-aural headphones for the manual test. Although thresholds obtained from two automated procedures showed greater variations at 8,000 Hz, compared with the manual test, there was no significant difference between the two automated thresholds. Therefore, it was considered that the variation at 8,000 Hz was due to the difference in transducer types.

Hearing thresholds obtained at 250 Hz also showed a greater variation between automated and manual audiometry. It was thought to be possibly due to the non-sound treated environments or to the suboptimal fitting of the insert earphones (Abu-Ghanem et al., 2016; Corry et al., 2017; Barbour et al., 2019; Sandström et al., 2020). However, this did not explain the variation at 250 Hz in this study. Both the manual and automated audiometry were tested in a soundproof room, and circumaural headphones were placed above the insert earphones to increase the attenuation of ambient sound when tested for automated audiometry. More than 40% of thresholds difference at 250 Hz in Group A were equal to 0 dB, compared with only 20% in Group S. It is possible that low-frequency tone is not easily recognized by human ears and requires more attention to obtain an accurate threshold. The ascending method used in Group A, which presented more tones than Group S, facilitated the reliable hearing threshold at 250 Hz. The difference at 250 Hz between the two automated procedures was an intriguing issue which could be explored in further research in the field of automated audiometry.

In this study, both the testing time and the total number of reactions in Group S were significantly lower than those in Group A. The long-time testing would also aggravate the uncomfortable feelings of the subjects. Participants who were more willing to accept the manual audiometry most had a longer testing time, and they felt ear stuffy from insert earphones or heaviness from earphone cups. In a previous study (Storey et al., 2014), subjects also reported discomfort from the weight and pressure of the headset over time. In this study, only air conduction thresholds were performed, and it would have taken longer if the bone conduction had also been measured.

Although the correlation was lower at low and high frequencies than at medium frequencies, these errors were still within acceptable limits when clinically explaining the test results. Therefore, in large-scale screening settings, or in some special populations, such as subjects with short attention spans, the shortened ascending method should be a better choice in automated testing. In mass screening and in areas with inadequate medical facilities, where the testing environment often does not meet the standard requirements. KUDUwave has been shown to obtain comparable results to manual testing in a free-field environment (Visagie et al., 2015), with the application advance in clinically heterogeneous populations (Brennan-Jones et al., 2016), and in bone-conduction test (Swanepoel de and Biagio, 2011), the automated audiometry device has great potential for service delivery in low- and middle-income countries and in rural and remote areas lacking medical facilities, which is an important direction for our future research.

## Study limitations and future directions

One of the limitations of this study is that the testing sequence of the manual and automated methods was not counter-balanced, the manual testing was conducted firstly, which could cause an order effect. Secondly, all subjects in this manuscript were with normal hearing, and the correlation between the results of the shortened/ascending and manual method was good, but further research is needed to determine whether the correlation is still accepted when the automated hearing test was conducted in people with different degrees of hearing loss. Thirdly, all the pure tone audiometry tests in this study were conducted in the sound booth, the subsequent studies need to be conducted to compare the hearing thresholds of subjects with different degrees of hearing loss in a non-isolated environment.

## Conclusion

In normal hearing subjects, there is a high correlation between automated and manual audiometry thresholds, but the variation was higher at 8,000 Hz. The test time was shorter using the shortened ascending method than the ascending method, but the accuracy of the two automated procedures differed statistically at 250 Hz. A more delicate threshold-seeking, the ascending procedure, may address this problem when testing low frequencies.

## Data availability statement

The raw data supporting the conclusions of this article will be made available by the authors, without undue reservation.

## Ethics statement

The studies involving human participants were reviewed and approved by the Beijing Tongren Hospital, Capital Medical University. The patients/participants provided their written informed consent to participate in this study.

## Author contributions

HL, BL, and XF designed the experiments. HL and BD carried out the experiments. HL, YW, and XF analyzed the experimental data. HL wrote the manuscript. XF, BL, and YW reviewed the manuscript. All authors contributed to the article and approved the submitted version.
