# Supplementary material for: Clinical comparison of two automated audiometry procedures
Source: Front Neurosci. 2022 Oct 11;16:1011016. doi: 10.3389/fnins.2022.1011016 (PMC9595274; doi:10.3389/fnins.2022.1011016)
Supplement: Supplementary file 1 [file Table_1.docx]

STable 1. Comparison of manual and automated audiometry protocols

|  | Manual procedure | Automated procedure |
| --- | --- | --- |
| Stimulus | Pure tone | Pure tone |
| Presentation duration | 1~2s | 1s |
| Subjects response | Pressing a signal switch | Pressing a signal switch |
| Correct response determination | Experience of the tester | Response within 2.5s after delivering pure tone |
| Initial intensity for the first frequency | 30dB HL | 30dB HL |
| Initial intensity for the subsequent frequencies | 10dB above the threshold of previous frequency | 30dB HL |
