# Supplementary material for: Clinical comparison of two automated audiometry procedures
Source: Front Neurosci. 2022 Oct 11;16:1011016. doi: 10.3389/fnins.2022.1011016 (PMC9595274; doi:10.3389/fnins.2022.1011016)
Supplement: Supplementary file 2 [file Table_2.docx]

STable 2: The effect of gender and age on the results of the automated test by ANOVA test.

| Characteristics |  |  | Difference of thresholds | Abs difference of thresholds |
| --- | --- | --- | --- | --- |
|  |  |  | *p* value | |
| Group A | n=50 |  |  |  |
| Sex | Male/Female | 23/27 | 0.658 | 0.256 |
| Age (M±SD) | Group 1(<30) | 20.9±3.4 | 0.704 | 0.466 |
|  | Group 2(>30) | 32.7±1.9 |  |  |
| Group S | n=50 |  |  |  |
| Sex | Male/Female | 21/29 | 0.730 | 0.955 |
| Age (M±SD) | Group 1(<30) | 23.5±3.6 | 0.444 | 0.441 |
|  | Group 2(>30) | 32.9±2.3 |  |  |

Difference of thresholds: the value of the difference between the manual and the automated thresholds (manual minus automated values); Abs difference of thresholds: the absolute value of the difference between the manual and the automated threshold.
